# An E-Nose for the Monitoring of Severe Liver Impairment: A Preliminary Study

**DOI:** 10.3390/s19173656

**Published:** 2019-08-22

**Authors:** Danila Germanese, Sara Colantonio, Mario D’Acunto, Veronica Romagnoli, Antonio Salvati, Maurizia Brunetto

**Affiliations:** 1 Institute of Information Science and Technology (ISTI), National Research Council (CNR), 56127 Pisa, Italy; 2 Institute of Biophysics (IBF), National Research Council (CNR), 56127 Pisa, Italy; 3 Gastroenterology and Hepatology Unit, University Hospital of Pisa, 56127 Pisa, Italy

**Keywords:** electronic noses, gas sensors, data processing, breath analysis, liver impairment, hepatic encephalopathy

## Abstract

Biologically inspired to mammalian olfactory system, electronic noses became popular during the last three decades. In literature, as well as in daily practice, a wide range of applications are reported. Nevertheless, the most pioneering one has been (and still is) the assessment of the human breath composition. In this study, we used a prototype of electronic nose, called Wize Sniffer (WS) and based it on an array of semiconductor gas sensor, to detect ammonia in the breath of patients suffering from severe liver impairment. In the setting of severely impaired liver, toxic substances, such as ammonia, accumulate in the systemic circulation and in the brain. This may result in Hepatic Encephalopathy (HE), a spectrum of neuro–psychiatric abnormalities which include changes in cognitive functions, consciousness, and behaviour. HE can be detected only by specific but time-consuming and burdensome examinations, such as blood ammonia levels assessment and neuro-psychological tests. In the presented proof-of-concept study, we aimed at investigating the possibility of discriminating the severity degree of liver impairment on the basis of the detected breath ammonia, in view of the detection of HE at its early stage.

## 1. Introduction

The liver plays a vital role in human body in maintaining metabolic balance. The loss of liver function causes significant damage to the body, as in the late stage of fibrosis of the liver, namely cirrhosis, when the damage to the organ becomes irreversible [1]. According to the World Health Organization (WHO), cirrhosis accounts for 1.8% of all deaths in Europe [2]. A serious complication of cirrhosis is Hepatic Encephalopathy (HE) [3,4].

HE is defined as a spectrum of neuropsychiatric abnormalities in patients with acute liver dysfunction (after exclusions of brain disease), with a negative impact on survival [5,6,7,8]. It is characterised by personality changes, confusion, disorientation, intellectual impairment and depressed level of consciousness [9]. Although HE exact pathogenesis is still an open issue, accumulation of ammonia in the systemic circulation and in the brain, from weak hepatic function and porto-systemic shunting, has been assessed as a primary factor [10].

Clinical testing for HE includes neurophysiologic tests such as the “trail making test” A and B (TMT-A and TMT-B), “block design test” and “digit-symbol test” (DST) [11]. Nevertheless, such tests can be very cumbersome for the patients and very time-consuming to be performed in the busy physician’s office. Also, the requirement for subjects to manually connect the numbers and the letters with a pencil line, adds a sort of inter-variability among the individuals due to a number of factors (e.g., presence of arthritis, handedness and dexterity in general) that are not related to the cognitive processes of interest. Moreover, sometimes the subjects do not fully understand the instructions of the tests [12]. These issues may lead to a complicated interpretation of TMT results.

Biochemical testing for HE may consist of blood ammonia level measurement, but it is not usually used as its accuracy may vary depending on specimen handling and measurement techniques [13].

Therefore, the lack of reliable examinations for the assessment of HE, and, more generally, the obtrusivity of the majority of the liver function clinical tests (from blood test to liver biopsy), are encouraging the scientific community to look for alternative diagnostic methods.

It has been well-established that the more the levels of ammonia in the blood increase, the more its concentration in the exhaled breath gases is [14,15,16,17]. Therefore, the possibility to use a real-time, non-invasive breath ammonia test system to detect liver dysfunction and evaluate its degree of severity (with particular attention to HE) would be a very positive step forward.

A solution may come from electronic noses.

It was 1982 when the term “electronic nose” was introduced in the scholarly literature after the development of the first e-nose by Persaud and Dodd [18]. In 1991, a research workshop was organized in Reykjavik, Iceland, which accelerated interest in the field. Since then, this kind of device attempts to emulate the human olfactory system by using an array of chemical gas sensors and a specific signal processing. Advances in sensor development, software innovations and progresses in microcircuitry design led to the invention of many types of e-noses, based on different detection principles and transduction mechanisms [19]. Such technological advances in the field of electronic olfaction has been closely correlated with the spread of new applications, e.g., from environmental gas monitoring, to the assessment of the quality of food, etc.

In recent years, the idea of using e-noses also for clinical applications has gained the attention of the scientific community [20]. Relying on the fact that different diseases have specific effects on a number of important key metabolic pathways, and the by-products of such processes travel in blood and, hence, participate to alveolar exchanges, the analysis of exhaled breath composition enables the observation of the biochemical processes that occur in human body in a non invasive way [21]. The idea is to exploit the earlier disease-detection capabilities of e-noses with the potential of accelerating the diagnostic process [22,23,24]. However, among the 3000 different volatile organic compounds (VOCs) that have been identified in human breath [25], the challenge is to capture the whole pattern of exhaled VOCs which can be seen as the volatile signature related to a particular underlying condition.

In the context of breath analysis, e-noses may serve as a cheap and rapid alternative to the standard chemical analytical methods (e.g., gas chromatography-mass spectrometry, GC-MS) [26]. E-nose devices for clinical applications are portable, lightweight, low-cost tools that provide real-time results. These instruments have proven, on one hand, to overcome the limitations of the standard instrumentation for gas analysis, and, on the other hand, to be able to detect a wide range of unique and often abnormal VOC-biomarker metabolites generated by many different types of diseases.

In many studies, they have been employed in different fields of medicine: in oncology, for instance, to identify lung cancer volatile signature [27], or to detect colorectal carcinoma [28,29], breast cancer [29,30], prostate tumour [29,31]; in infectiology [32]; in respiratory medicine to evaluate asthma [33] or to discriminate between healthy subjects and patients suffering from chronic obstructive pulmonary disease (COPD) [34]; to early detect gastro-intestinal diseases [22]; to diagnose renal dysfunction [35,36]; to monitor diabetes [37,38]; to detect neuro-degenerative disorders such as Alzheimer’s and Parkinson’s disease [39]. In addition to preventative healthcare applications, e-nose devices may be also useful for therapeutic applications, e.g, to monitor the response to drugs [40].

However, there are few studies on patients with chronic liver disease with e-nose [41].

A large number of methods that were developed for detecting and measuring ammonia in human breath and discriminating between healthy and cirrhotic subjects involve techniques such as ion flow tube mass spectrometry (SIFT-MS), or GC-MS [42], or photoacoustic laser spectrometry (PALS) [43,44], or proton transfer reaction time-of-flight mass spectrometry (PTR-MS) [45]; although very accurate, such laboratory tools are not portable, very expensive, and far from being translated into daily clinical practice, where the major goal is to provide easy-to-use point-of-care systems.

For this purpose, several micro-systems were developed. One of them is described in [46]. In this work, Timmer and colleagues present a micro-fluidic system able for detecting ammonia by using a conductivity sensor. Nevertheless, such method may be time consuming as ammonia needs to be converted to ammonium and, then, back to gaseous ammonia for measurement. In [47], Toda et al. detected breath ammonia by dissolving it in a droplet of concentrated sulfuric acid on the top of a conductivity sensor. In general, although accurate, the daily application of such types of methods may be very laborious, due to the difficulty of their replication.

Other systems able to detect ammonia involve the use of optical sensing systems. Although very sensitive to gaseous ammonia in the part-per-billions (ppb) or part-per-trillions (ppt) regime, they demonstrated to require expensive set-up, as reported in [48].

Zan et al. [49] developed a pentacene-based organic thin-film transistors (OTFT), whose sensitivity was improved by an UV irradiation treatment which modified the functional groups on the poly (methyl methacrylate) dielectric layer. OTFTs are inexpensive and manageable diagnostic device because of their low-cost manufacturing process [50]. In addition, they show good sensitivity to gaseous compounds. Indeed, the device developed by Zan and colleagues was able to detect 0.5 up to 5 part-per-millions (ppm) concentration of gaseous ammonia, which is the critical range of breath ammonia levels that can discriminate between healthy subjects and patients with cirrhosis or renal impairment. Nonetheless, such a system was tested only in the laboratory setting and not on a population.

Other studies aimed at developing nanomaterial-based sensors selective to ammonia. For instance, Gouma and co-workers [51] developed a $MoO_{3}$-based nanosensor able to selectively measure concentrations of ammonia gas at ppb levels. Nevertheless, also in this case, the sensor was tested only in a breath-simulating environment. AmBeR${}^{®}$, a breath ammonia measurement device, was designed and developed by BreathDX (http://https://www.breathdx.com/), a UK-based company founded by Prof. T. Killard. The device is based on array of very sensitive gas sensors fabricated using inkjet-printed functional nanomaterials, capable of ppb limits of detection [52]. Nevertheless, AmBeR${}^{®}$ was designed to diagnose and manage a range of conditions (from stomach ulcer to chronic liver disease, to non-invasive volume drug toxicity study), thus it is not specific to monitor chronic liver impairment.

In this paper, we present a proof-of-concept study which aimed at detecting ammonia in the breath of a population of subjects suffering from chronic liver impairment. For this purpose, we used a portable, low-cost, easy-to use electronic nose, which, in contrast to the standard techniques for gas analysis (e.g., GC-MS), allowed for assessing human breath composition in real time. The device was tested on a population of 64 subjects, among which 16 healthy individuals, 20 subjects with chronic liver impairment, 22 cirrhotics and six cirrhotics with recent episode of HE. Our work, focusing only on liver impairment, went beyond the state of the art as it was not limited in discriminating healthy from diseased subjects, but laid the foundation for a more detailed study aiming at developing a method to discriminate the severity degree of liver impairment on the basis of the detected breath ammonia.

## 2. Materials and Methods

### 2.1. The Wize Sniffer

**Figure 1:**
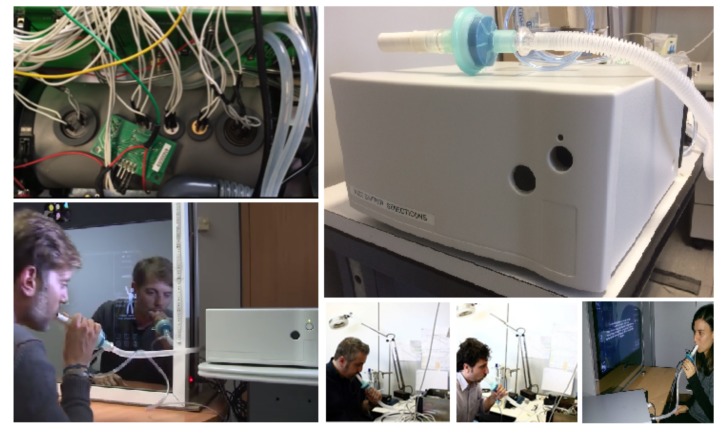
The Wize Sniffer (WS). On the top, left: the gas sensors in the gas sampling chamber are shown. On the top, right: WS external configuration (WS dimensions: 30 cm × 30 cm × 14 cm). Others: the WS while performing a breath test.

In the present work, we used a prototype of e-nose (shown in Figure 1) purposely designed for the evaluation of human breath composition. Its name is Wize Sniffer (WS) and it was developed for user’s health self-monitoring and self-surveillance, also in home environment [53,54].

The core of the WS is the signal acquisition module, that is composed of three elements: (i) the gas sampling chamber, (ii) the gas sensor array and (iii) the micro controller board.

The gas sampling box was made up of acrylonitrile-butadienestyrene (ABS) and Delrin, which are two materials that do not interfere with sensors sensitivity, and its capacity is 600 mL according to the human resting tidal volume [55].

Within the gas sampling chamber, six metal oxide semiconductor (MOS) gas sensors, manufactured by Figaro Engineering (Osaka, Japan, https://www.figaro.co.jp/en/), were placed.

The employed gas sensors (listed in Table 1) showed long term stability and reproducibility of gas response, great chemical stability of the sensing material, high sensitivity to target VOCs, short response and recovery time, small dimensions, compactness and low cost [56].

Nonetheless, their behaviour was strongly influenced by humidity. Indeed, in Table 1, sensor drift due to variations in humidity is reported. The computation of sensor drift coefficients, as well as the experimental tests aiming at studying the gas sensors’ behaviour are reported in more detail in [57,58]. However, humidity variations were monitored and evaluated in real time, during each breath test, by means of a sensor for temperature and humidity (Sensirion SHT11) which was integrated into the gas sampling box. In addition, a heat and moisture (HME) disposable filter, made of hygroscopic material, was placed in series to the disposable mouthpiece of the WS in order to absorb the majority of the water vapour present in exhaled breath. A HME filter also allows for holding users’ oral bacteria.

The exhaled gases, after passing through the disposable mouthpiece and the HME filter, flow into a corrugated tube, made of polyvinyl chloride (PVC), and reach the gas sampling chamber. A flowmeter allows for assessing in real time user’s flow rate and for calculating the exhaled gas volume. In addition, a sampling pump injected, at a fixed rate (120 mL/s), the sampled exhaled gas to other two sensors, which have faster response time and work in flowing-regime. They detected oxygen and carbon dioxide and were respectively based on an electrochemical cell and an infrared source.

A signal conditioning module (mainly composed of a set of voltage buffer amplifiers LM124-N, Texas Instrument, Dallas, TX, US) read and filter sensor raw outputs and transfer them from the acquisition module to a widely employed open source controller board: an Arduino, Interaction Design Institute, Ivrea, Italy Mega2560.

Finally, in order to facilitate sensors recovery time, a flushing pump “purges” the gas sampling box with ambient air after each breath test.

### 2.2. Data Pre-Processing

Pre-processing methods are strongly related to the underlying sensor technology: on one hand, they aim at compensating the influencing factors which affect gas sensor response; on the other hand, they aim at extracting robust descriptive features from sensor signals for further analysis.

In the WS, Arduino Mega2560 reads and pre-processes sensor outputs in real time.

First, on the basis of flow-meter output, the exhaled volume was calculated. If it was lower than 600 mL (that is, gas sampling chamber’s volume), the breath test must be repeated because the exhaled volume was not sufficient to perform the analysis. Otherwise, gas sensor outputs were pre-processed.

For this purpose, the baseline is manipulated. Three baseline manipulation methods are commonly used [59]: (i) the difference method subtracts the baseline and it is used to eliminate additive drift from sensor response; (ii) relative manipulation divides by the baseline, remove multiplicative drift and generates dimensionless response; (iii) fractional manipulation subtracts and divides by the baseline, generating a dimensionless and normalised response. In the case of the WS, the fractional method for baseline manipulation is used, as it removes both additive and multiplicative errors.

In addition, a set of features were extracted from each sensor output signal. Feature extraction method is one of the key-points of performance improvement of an e-nose systems [60]. Here, for each sensor output signal, three features are extracted: (i) the value at curve plateau ($\Delta X_{s}\left(\infty \right)$), as it represents the steady state of the entire dynamic response process [60,61,62,63]; (ii) the response time ($T_{r}$), as it is characteristic of each vapour/sensor pair, concentration independent and shows high repeatability [64]. It is measured from 20 to 75% of $X_{s}\left(t\right)$; (iii) the maximum slope of the curve ($dX_{s}\left(t\right)/dt$).

Also humidity values (within the gas sampling chamber) were read before and after each breath test, in order to eventually compensate sensors drift due to humidity.

### 2.3. Experimental Tests

In the present proof-of-concept study, we exploited the potentialities of the WS to: (i) detect ammonia in the breath of patients suffering from chronic liver impairment; (ii) to investigate the possibility of discriminating the severity degrees of liver impairment on the basis of the detected breath ammonia.

The study included 64 subjects; 20 women (mean age: 52) and 44 men (mean age: 55) among which 20 non-cirrhotics with chronic liver disease (NC-CLD), 22 cirrhotics (CIRRH), six cirrhotics with recent episodes of HE (CHE) and 16 healthy controls (HC). The diagnosis of cirrhosis was based on liver biopsy or on clinical, biochemical and ultrasonographic tests. The presence of portal-systemic shunts was assessed by US and CT scan. The Child–Pugh and the model for end-stage liver disease (MELD) scores were calculated. A blood sample was drawn for each subject for a complete blood count, prothrombin time (PT), bilirubin, international normalized ratio (INR), and liver panel, albumin and creatinine determinations. The presence and the degree of HE were evaluated by focused neurologic exams and psychometric tests, including TMT-A and TMT-B and DST [65]. Exclusion criteria were: alcohol/psychoactive drugs at baseline, neurological disease, lack of compliance with psychometric evaluation, chronic illness (e.g., cardiac or renal insufficiency, diabetes, COPD).

Regarding the breath tests, there is no consensus in literature about how measurement of ammonia in exhaled breath should be measured [17]. In this study, the mixed expiratory breath sampling technique [66,67,68] was used, given its easily manageable and cost-efficient applicability. The volunteers were required to: (i) first, take a deep breath in; (ii) then, hold the breath for 10 s; (iii) finally, breath out once through the WS mouthpiece trying to keep the expiratory flow low (about 160 L/min ± 10%) and constant, and to completely empty their lungs. All the participating subjects were under the same conditions of environmental temperature and humidity when breath test was performed, in a seated position, at morning, fasting and several hour after brushing their teeth.

Breath carbon dioxide was monitored in real time, as its profile defines the quality of the breath sample [26], as well as the breath flow rate. The breath ammonia was detected with TGS2444 and TGS2602 gas sensors of the WS gas sensor array (see Section 2.1). After each breath test, the gas sampling chamber was “purged” with ambient air by means of the flushing pump.

The tests were conducted with the support of the Hepatology Unit of the University Hospital of Pisa (protocol n. 5643—Scientific Collaboration Agreement between the Institute of Information Science and Technologies of the National Research Council, Pisa, Italy and the Department of Clinical and Experimental Medicine, University of Pisa, Pisa, Italy). The methods and the protocol were submitted to the Ethical Committee of the Azienda Ospedaliero Universitaria Pisana for approval. The use of the electronic nose was approved by the E.C. of Area Vasta Nord–Ovest Azienda Ospedaliero Universitaria Pisana, Pisa, Italy (protocol n. 54625) and the Ethics and Bioethics Committee of the National Research Council of Italy, Rome, Italy (protocol n. 86129). All the subjects provided signed informed consent before enrolment.

## 3. Results

As reported in Section 2.1, for this study a sub-group of the sensors embedded in the WS were used: TGS2602 and TGS2444, sensitive to ammonia.

All the data were downloaded onto a database designed for the study in the format of Microsoft Excel 2007 (Microsoft Corporation, Seattle, WA, USA). Statistical analysis was performed in MATLAB${}^{®}$ and R${}^{®}$ (version 3.2.4) environments. First, descriptive statistics were used to quantitatively describe and summarise breath data. In Table 2 the features extracted from TGS2444 and TGS2602 output curves (see Section 2.2) are reported. For each class of subjects (HC, NC-CLD, CIRRH, and CHE) the median value (and the relative inter-quartile range IQR) of each parameter is reported.

As can be noted, the used Taguchi gas sensors (TGS2444 and TGS2602) gave good results in detecting breath ammonia. In particular, sensor maximum outputs increased with increasing liver impairment, as we expected. The median value of TGS2444 maximum output in HC was 0.39 V (IQR: 0.14 V), in NC-CLD patients it was 0.63 V (IQR: 0.41 V) (*p*-value: 0.004; the statistical significance threshold of 0.05 was corrected (lowered) for multiple comparisons. In this case we used Bonferroni correction), in CIRRH subjects it was 0.76 V (IQR: 0.58 V) (*p*-value: $3.155\times 10^{-4}$) and in CHE patients it was 1 V (IQR: 0.74 V) (*p*-value: $2.711\times 10^{-4}$). The median value of TGS2602 maximum slope in NC-CLD patients was 0.06 (IQR: 0.05), in CHE subjects it was 0.11 (IQR: 0.17) (*p*-value: 0.007). The median value of TGS2602 maximum slope in CIRRH patients was 0.03 (IQR: 0.02), in CHE subjects it was 0.04 (IQR: 0.06) (*p*-value: 0.004). The difference of the sensor features median values between NC-CLD and CIRRH patients did not result statistically significant.

**Figure 2:**
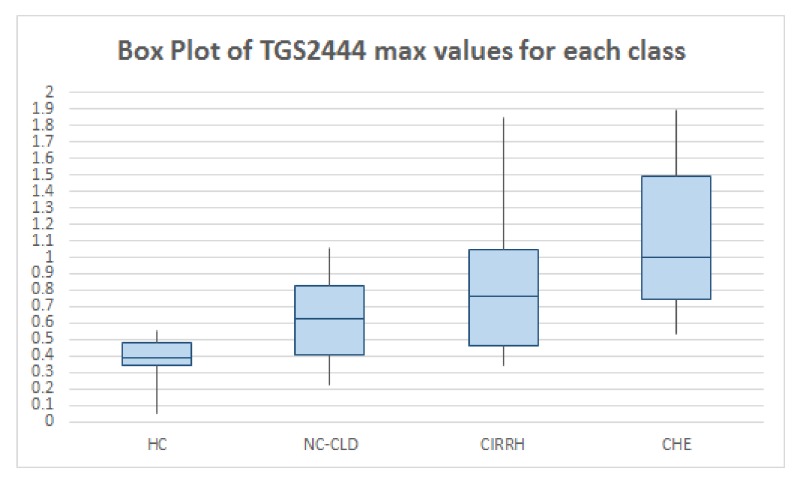
Box plot representing the median values, first quartile and 3rd quartile of TGS2444 maximum outputs relative to healthy controls (HC), non cirrhotic-chronic liver disease (NC-CLD), cirrhotics (CIRRH) and cirrhotics with a recent episode of Hepathic Hencephalopathy (CHE).

**Figure 3:**
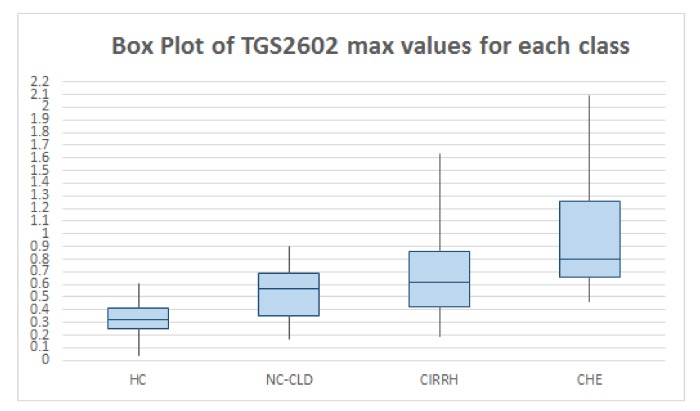
Box plot representing the median values, first quartile and 3rd quartile of TGS2602 maximum outputs relative to HC, NC-CLD, CIRRH and CHE.

**Figure 4:**
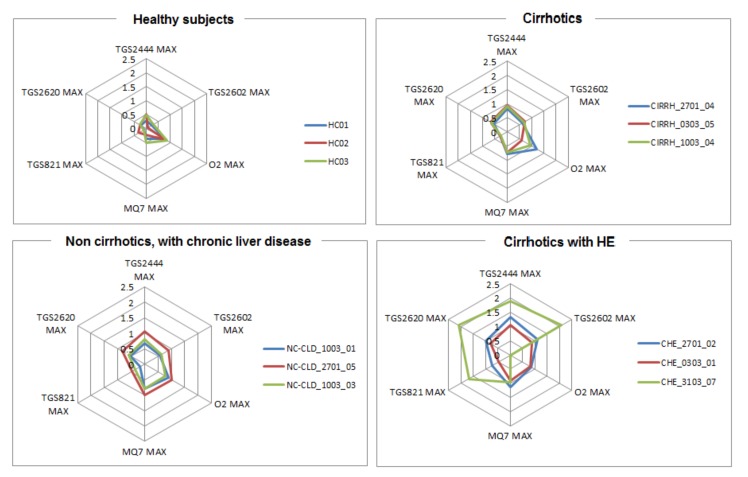
Comparison of radar plot profiles relative to HC, NC-CLD, CIRRH and CHE. Radar plots showed a concordant rise in value for TGS2444 and TGS2602 (sensitive to ammonia) maximum output, from HC to CHE subjects. However, a change in the whole sensors’ outputs pattern can be observed.

The results reported in Table 2 can be graphically observed also in Figure 2 and Figure 3. In addition, in Figure 4 the outputs relative to all the WS sensors are shown for three subjects taken, just as example, from each class. Visual analysis of these radar-plot profiles showed a progressive concordant rise in value for TGS2444 and TGS2602 maximum output, from healthy to cirrhotics with HE subjects. However, a change in the whole sensors output pattern was observed. Indeed, cirrhotics with HE showed, in general, a wider radar plot profile.

**Figure 5:**
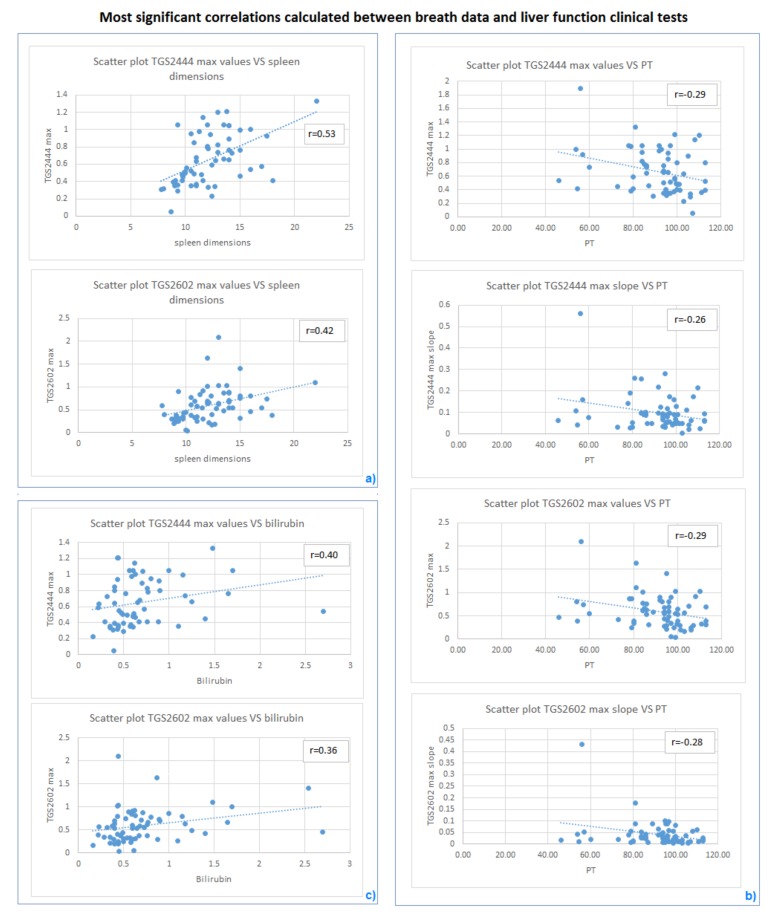
Most significant correlations calculated between breath data (TGS2444 and TGS2602 outputs) and liver function clinical parameters. The scatter plots visually show the relationship between (**a**) subjects spleen dimensions and ammonia sensors maximum values; (**b**) prothrombine time (PT) and ammonia sensors outputs (maximum value and maximum slope); (**c**) bilirubin and ammonia sensors maximum values.

Then, a bivariate analysis allowed for quantitatively describing the relationship between breath data and liver function tests. In particular, Pearson’s correlation coefficient ρ was calculated between the variables. Spleen dimensions showed significant positive correlation with both TGS2444 and TGS2602 maximum output (ρ = 0.53 *p*-value = 0.0001939 and ρ = 0.42 *p*-value = 0.001814, respectively; a value of *p*< 0.05 was considered to be statistically significant), as shown in Figure 5a). Negative correlations were found between PT and TGS2444 maximum output (ρ=−0.29*p*-value = 0.02785), TGS2444 maximum slope (ρ=−0.27 *p*-value = 0.0336), TGS2602 maximum output (ρ=−0.30 *p*-value = 0.01767), TGS2602 maximum slope (ρ=−0.29 *p*-value = 0.02057), as shown in Figure 5b). Also, TGS2444 maximum output (ρ = 0.40 *p*-value = 0.01294) and TGS2602 maximum output (ρ = 0.36 *p*-value = 0.02569) showed positive correlation with serum bilirubin, as shown in Figure 5c).

Firstly, given that sensor outputs coherently increased with the severity of liver function impairment (Table 2) and, secondly, significant correlations between sensor outputs and a set of liver function-related parameters, a further step consisted of evaluating WS diagnostic capability by means of reciever operating characteristic (ROC) curves analysis. We looked for cut-off values in sensor output features that allowed to differentiate healthy subjects from patients with liver disease, and, among the latter, those with and without cirrhosis. In addition, among the cirrhotics, sensors cut-off values were looked for to differentiate those with and without recent episode of HE (even though the number of CHE was low).

**Figure 6:**
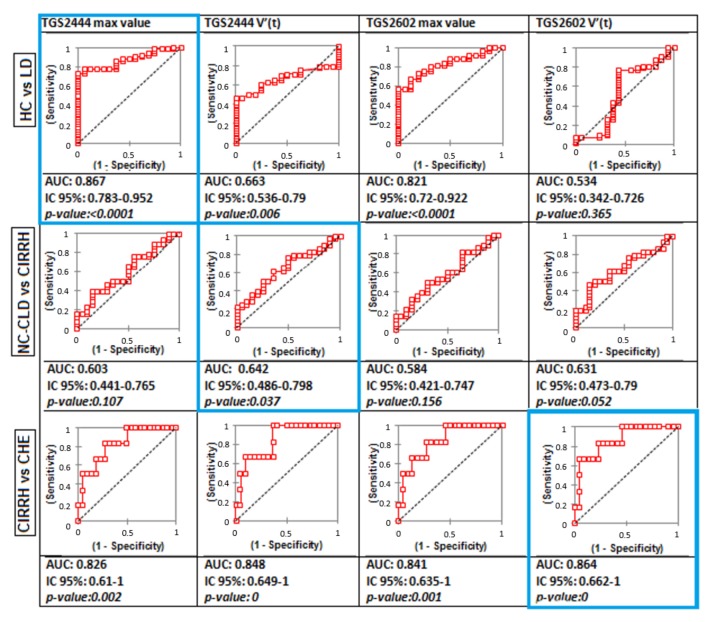
Comparison of receiver operating characteristic (ROC) curves for TGS2444 and TGS2602 output features (maximum output and first derivative) in the total evaluated population (first row, HC versus subjects with liver disease LD), in the population of patients with liver impairment (second row, NC-CLD patients versus CIRRH), in the population of cirrhotic patients (third row, CIRRH versus CHE).

A TGS2444 maximum value of 0.572 V permitted to differentiate healthy subjects from patients with liver disease in general (HC versus LD); indeed, the wider area under the curve (AUC)-ROC, as shown in Figure 6, was the one relative to TGS2444 maximum output (AUC = 0.867, 95%CI: 0.783–0.952, *p*-value: < 0.0001).

Among the patients with liver impairment, the boundary between subjects suffering from chronic liver disease with (CIRHH) and without (NC-CLD) cirrhosis was more difficult to establish. Low values of AUC can be observed for TGS2444 and TGS2602 maximum value. The wider AUC can be observed for TGS2444 maximum slope (AUC = 0.642, 95%CI: 0.486–0.798, *p*-value: < 0.037): a value of 0.093 discriminated between cirrhotic and non-cirrhotic with chronic liver impairment patients.

In cirrhotic patients, the boundary between cirrhotic with (CHE) and without (CIRRH) HE was more clear. Indeed, as widely reported before, the hyperammonemia in patients with HE is more pronounced. A value of 0.065 for TGS2602 maximum slope (AUC = 0.864, 95%CI: 0.662–1, *p*-value = 0) permitted to differentiate between cirrhotics with and without HE.

A summary of these results is reported in Table 3.

## 4. Discussion and Conclusions

In the present paper, we reported a preliminary study aiming at detecting ammonia in the breath of patients suffering from chronic liver disease. The experimental tests included a population of 64 subjects, among which 16 healthy individuals, 20 non-cirrhotic patients with chronic liver impairment, 22 cirrhotics and six cirrhotics subjects with recent episodes of HE.

In literature, the majority of the studies aiming at detecting ammonia in human breath used standard instrumentation for gas analysis (e.g., GC-MS) [42]. Such tools are very accurate, but they are very far to be exploited into daily clinical practice, as they are very expensive and time consuming [40]. In addition, they can be used only by qualified personnel. Here, we used an easy-to-use, low-cost electronic nose, named Wize Sniffer able to evaluate human breath composition in real time.

In addition, most of the studies on the detection of breath ammonia focused on the discrimination between healthy subjects and cirrhotic patients [16,17,42] Although our work did not include measurements of blood-ammonia levels, neither the exact assessment of breath ammonia concentration levels, it demonstrated that the WS permitted to discriminate well not only between healthy subjects and patients with liver impairment, but also between cirrhotics with and without HE.

By using both the dynamic and the steady state features of two MOS-based gas sensors sensitive to ammonia, we obtained a picture about systemic ammonia levels. Indeed, we showed that:

- the used MOS gas sensors gave good results in detecting breath ammonia, also at <ppm levels;
- the median values of the features extracted from sensor signals increased with increasing liver impairment;
- significant correlations were found between gas sensor features and a set of standard liver function parameters (e.g., PT, bilirubin, spleen dimensions);
- cut-off values were found in gas sensor features which permitted to discriminate between the several group of individuals (from HC to CHE subjects).

Along with such promising results, our work has several limitations. First of all, the limited number of involved subjects, especially considering the number of individuals for each group.

However, this preliminary study aimed at laying the foundations for a larger one. Indeed, in the immediate future, we intend to include a larger number of patients to confirm, extend and enhance the results here presented. A larger number of recruited subjects, including patients with suspected liver impairment, will allow us for implementing a learning algorithm able to recognise the severity of liver impairment, based on the detected breath ammonia, and eventually detect HE at its early stage, namely minimal HE (MHE) or sub-clinical HE. MHE is the mildest form of spectrum of HE and has attracted increasing attention. Typically, patients with MHE have no recognisable clinical symptoms of HE (both the patient and those around the patient, including physicians, are not aware that such condition is present) but have mild cognitive and psychomotor deficits [69]. As MHE predicts the development of HE, it is very important to detect such condition and promptly give the patient ammonia-lowering agents (i.e. lactulose and probiotics). The diagnostic criteria for MHE have not been standardised but normally it is detected by means of the specific but very time consuming neurophysiologic tests above mentioned (TMT-A, TMT-B and DST) [70]. A valid help may come through the use of a very rapid diagnostic system that exploits unobtrusive, non-invasive techniques, such as e-noses.

Another limitation of our study relied on the lack of standardised procedures for breath ammonia sampling. In our study we used the mixed expiratory breath sampling, which considers the whole volume of exhaled breath, given its easy manageability and cost efficient applicability. In addition, carbon dioxide profile was continuously monitored during each breath test to evaluate the quality of the breath sample, as well as the volume flow rate. Nevertheless, the lack of standardised guidelines does not allow breath samples collected and analysed in different laboratories to be compared [26].

Finally, our *proof of concept* study also demonstrated the unobtrusiveness, the safety and the discriminative properties of the WS. However, in future we also aim at enhancing the performances of the WS itself, boosting the selectivity and the specificity of both the measurement and the signal processing modules. Some of the improvements may include:

- the design of a new gas sampling box, with a more suitable geometrical shape to ensure all of the gas sensors receive the same amount of air flow during each breath test [71];
- the use of new materials for the gas sampling box, e.g., organic tehermoplastic polymers such as PEEK (Polyether ether ketone) [72], to be sure to avoid any absorption phenomenon of volatile molecules on the internal surface of the gas sampling box itself;
- a system based on a solenoide valve to automatically sample the portion of exhaled volume of interest;
- the integration of a controller board with higher computing power.

E-noses systems with high disease specificity and sensitivity may offer a highly impactful solution for the early detection of a range of diseases as well as a valid tool for treatment response self-assessment, thus significantly contributing to the personalised medicine approach.

## Figures and Tables

**Table 1:** Metal oxide semiconductor (MOS)-based gas sensors embedded into Wize Sniffer (WS) acquisition module.

Sensor	Detected Molecules	Best Detection Range (ppm)	Drift Coeff. due to Humidity (ΔV / Δ hum (mV))
MQ7	carbon monoxide	20–200	296
	hydrogen	20–200	
TGS2620	carbon monoxide	50–5000	60
	hydrogen	50–5000	
	ethanol	50–5000	
TGS2602	ethanol	1–10	82
	hydrogen sulfide	1–10	
	hydrogen	1–10	
	ammonia	1–10	
TGS821	hydrogen	10–5000	120
TGS2444	ammonia	0.1–30	84
TGS4161	carbon dioxide	0–4000	56

**Table 2:** Median values of TGS2444 and TGS2602 max value, respose time and maximum slope for each class: healthy controls (HC), non cirrhotic-chronic liver disease (NC-CLD), cirrhotics (CIRRH) and cirrhotics with a recent episode of Hepathic Hencephalopathy (CHE). Within the brackets, also the inter-quartile range (IQR) is reported.

	TGS2444	TGS2444	TGS2444	TGS2602	TGS2602	TGS2602
	$\Delta X_{s}\left(\infty \right)$ (V)	$T_{r}$ (msec)	${\mathrm{dX}}_{s}\left(t\right)/\mathrm{dt}$ max	$\Delta X_{s}\left(\infty \right)$ (V)	$T_{r}$ (msec)	${\mathrm{dX}}_{s}\left(t\right)/\mathrm{dt}$ max
	(IQR)	(IQR)	(IQR)	(IQR)	(IQR)	(IQR)
HC	0.39 (0.14)	750 (250)	0.06 (0.03)	0.32 (0.16)	1250 (1562.50)	0.01 (0.07)
NC-CLD	0.63 (0.41)	1250 (625)	0.06 (0.05)	0.57 (0.34)	3750 (1125)	0.02 (0.01)
CIRRH	0.76 (0.58)	1000 (500)	0.09 (0.11)	0.62 (0.44)	2750 (1500)	0.03 (0.02)
CHE	1 (0.74)	750 (500)	0.11 (0.17)	0.8 (0.6)	2750 (1375)	0.04 (0.06)

**Table 3:** The cut-off sensor features which permitted to discriminate between HC versus liver disease (LD); in the population of patients with liver impairment, CIRRH versus NC-CLD; in the population of cirrhotic patients, CHE versus CIRRH.

	CUT-OFF	AUC-ROC	*p*-Value	VP	VN	FP	FN	SENS.	SPEC.
		*95%CI*							
HC	$TGS2444_{max}$ = 0.572 V	0.867	<0.0001	37	15	1	11	0.771	0.938
vs. LD		*0.783–0.952*							
NC-CLD	$TGS2444_{maxslope}$ = 0.093	0.642	<0.037	17	13	7	11	0.607	0.650
vs. CIRRH		*0.486–0.798*							
CIRRH	$TGS2602_{maxslope}$ = 0.065	0.864	0	4	21	1	2	0.666	0.954
vs. CHE		*0.662–1*							

## Abbreviations

The following abbreviations are used in this manuscript:

ABS	Acrylonitrile Butadiene Styrene
AUC-ROC	Area Under the Curve-Receiver Operating Characteristic
CHE	Cirrhotics with recent episode of HE
CIRRH	Cirrhotics
COPD	Chronic obstructive pulmonary disease
CT	Computer tomography
DST	Digit-simbol test
GC-MS	Gas chromatography-mass spectrometry
HC	Healthy controls
HE	Hepathic Hencephalopathy
HME	Heat and moisture exchanger
INR	International normalized ratio
LD	Liver disease
MELD	Model for end-stage liver disease
MHE	Minimal HE
MOS	Metal oxide semiconductor
NC-CLD	Non cirrhotic-chronic liver disease
OTFTs	Organic thin-film transistor
PALS	Photoacustic Laser Spectrometry
ppb	part-per-billions
ppm	part-per-millions
ppt	part-per-trillions
PT	Prothrombine time
PTR-MS	Protron transfer reaction time-of-flight mass spectrometry
PVC	Polyvinyl chloride
SIFT-MS	Selected ion flow tube-mass spectrometry
TMT-A	Trail-making-test A
TMT-B	Trail-making-test B
US	Ultrasound
VOC	Volatile organic compound
WHO	World Health Organization
WS	Wize sniffer
